# CONSOLATION SPACES: DO THEY HELP TO MAKE SERIOUS ILLNESS, DEATH, AND LOSS DISCUSSABLE IN THE COMMUNITY?

**DOI:** 10.1093/geroni/igad104.0389

**Published:** 2023-12-21

**Authors:** Sarah Dury

**Affiliations:** Vrije Universiteit Brussel, Brussels, Brussels Hoofdstedelijk Gewest, Belgium

## Abstract

A recent groundswell of movements and organisations is rising in Flanders, Belgium, stimulating engagement with serious illness, death, and bereavement. One result is the huge growth of public consolation spaces. However, it remains largely unknown how consolation spaces are developed, what actions are taken or how they are used. To investigate the development, meaning, their role in a neighbourhood, and the use of consolation spaces, a multi-method design was used. We first examined six different consolation spaces through focus groups. Various stakeholders took part in these, such as volunteers, members of the municipal administration, visitors to the consolation space and care staff. At the same time, an online survey aimed at the use of consolation spaces ran (N=123). Following, the consolation space initiators completed an online survey measuring specific project characteristics, observed outcomes and elements crucial to the realisation of the project (N=still running). Preliminary results indicate that consolation spaces are often created in collaboration with several organisations. The meaning of consolation spaces for people is a space where people seek silence, comfort or to feel connected. Most take or would like to take part in connecting activities linked to the consolation spaces such as consolation walks, reflection moments, planting flower bulbs, etc. People mainly visit this place alone or with a friend and this mainly on an annual basis. The connection with the neighbourhood is present within many consolation spaces. The discussion aims to critically reflect on the findings from the perspective of sustainability and systemic change within neighbourhoods.

